# New Avenues of Research for Nanoparticle Drug Delivery Systems

**DOI:** 10.3390/nano12234141

**Published:** 2022-11-23

**Authors:** Rafael Prado-Gotor

**Affiliations:** Department of Physical Chemistry, Faculty of Chemistry, University of Seville, C/Profesor García González 1, 41012 Seville, Spain; pradogotor@us.es

Knowledge of the different elements that determine the optimal method for drug loading and delivery nanosystems using nanoparticles of different natures is experiencing a remarkable boom in many scientific fields, especially in medicine, chemistry, biology, materials science and molecular biotechnology. Not only is it necessary to fully understand what type of nanoparticle is most appropriate in each particular situation, but the interactions of the used nanoparticle with the system under study or the analysis of the release process are important as well. To design nanoparticle drug delivery agents well, a high ratio of drug to nanoparticles must be achieved. Furthermore, biocompatibility and knowledge of the time the release process takes are fundamental factors for the construction of operative NPs as drug delivery vectors.

This Special Issue, “New Avenues of Research for Nanoparticle Drug Delivery Systems”, brings together recent research articles published in *Nanomaterials*. Specifically, five original research articles, one of them a review, were published by authors from different countries on what is a current issue in this field of research.

S. E. Kim et al. [1] prepared and characterized lactoferrin (LF) conjugated carboxylated-nanodiamonds (cNDs) in order to analyse their possible effects against oxidative stress, inflammatory response and osteogenic differentiation of cells. LF-NDs not only markedly suppress reactive forms of oxygen (ROS) in cells, but they also shelter cells in ROS environments and can significantly decrease the levels of pro-inflammatory cytokines (IL-1β and TNF-α) secreted by cells challenged with LPS. Furthermore, LF-NDs promote osteogenic differentiation of MC3T3-E1 cells by increasing alkaline phosphatase activity and calcium deposition via LF release.

L. Gómez-Segura et al. [2] investigated the ex vivo permeation of carprofen (CP) 2-(6-chlorocarbazole) propionic acid across different classes of porcine mucous membranes (buccal, sublingual and vaginal) and ophthalmic linings (cornea, sclera and conjunctiva) to differentiate between the CP-NP formulation and a CP solution (CP-Solution). The results showed that CP-NPs provide advantageous situations in most tissues. The structure of the material is not modified, being more effective and safer than the CP solution. This research opens the door to successfully test in situ treatments of many inflammatory diseases in animals or people.

P. M. Castillo et al. [3], working in the field of disease detection using nanosystems, have carried out a new method to determine urinary lysozyme content within a concentration range that is associated with monocytic and myelomonocytic leukemia, among other diseases. The authors describe a method based on obtaining CIELab parameters described by the CIE. The advantages of the method involve a very low cost and an extremely short detection time. In addition to being fast (less than 10 min) and economical, the described method does not require specialized personnel in the knowledge of specific analytical techniques. The required equipment implies having a commercial spectrophotometer or colorimeter, and a positive case can even be detected through an evaluation of the color with the naked eye with a reference solution. 

A. Gomes et al. [4] have developed an exhaustive study of the interaction energy between lysozyme and the surface of gold nanoparticles surrounded by citrate ions, considering with special attention those factors that can directly influence the performance of colloidal gold systems for the detection of the protein. Specifically, authors have analysed the stability of colloidal gold solutions, the influence of the diameter of the nanoparticles and the correct way to express the concentration of gold in the nanosystem to obtain the best and most accurate results. They found that the state of saturation implies an average number of 55 Lys per gold nanoparticle. On the other hand, it has been found that the free energy (ΔG^0^) corresponding to the interaction of the protein with the 10 nm gold surface is about −40.8 kJ mol^−1^.

B. Begines et al. [5] have prepared a review based on polymeric nanoparticles for drug delivery. This review focuses on the most recent applications and advances of nanoparticulate polymeric formulations as nanocarriers, especially those used to battle specific diseases. Studies about the use of different materials as nanocarriers should meet important requisites such as biocompatibility, biodegradability and non-immunogenicity. The toxicity associated with numerous drugs and classical galenic formulations or the complexity to treat diseases have progressed the prompt development of new alternatives to drug-eluting nanosystems. In this sense, polymers are macromolecules synthetized via a covalent union of one or different monomers that possess at least two functional groups, where they can react easily to constitute a chain to attain specific properties. Polymeric nanoparticles are not only pharmaceutical entities that may exhibit all the above-mentioned characteristics: their rich synthetic versatility allows them to be greatly customized to accomplish the final requirements. Particularly, polymeric nanoparticles for ocular drug delivery, for cancer diagnosis and treatment, as well as nutraceutical delivery, have been described in detail, as well as an interesting discussion concerning the future prospects of these systems.

I would like to thank all the authors and reviewers of this Special Issue. I also acknowledge the Assistant Editor, Riven Yang, for her trust, support and effort in moving this issue forward. In addition, authors are encouraged to submit original research articles and reviews in the next Special Issue: “Recent Advances in Targeted Therapy Using Multifunctionalized Gold Nanoparticles”.

## Data Availability

Not applicable.
